# Occupational exposure to noise and cold environment and the risk of death due to myocardial infarction and stroke

**DOI:** 10.1007/s00420-019-01513-5

**Published:** 2020-01-08

**Authors:** Hans Pettersson, David Olsson, Bengt Järvholm

**Affiliations:** Department of Public Health and Clinical Medicine, Section of Sustainable Health, Umeå University, 901 87 Umeå, Sweden

**Keywords:** Mortality, Ischemic heart disease, Cerebrovascular disease, Prospective cohort study, Work environment

## Abstract

**Purpose:**

The present study examined a possible association between occupational exposure to noise, working and living in cold conditions, and the risk of mortality in myocardial infarction and stroke.

**Methods:**

The present cohort study consists of 194,501 workers in the Swedish construction industry that participated in health examinations between 1971 and 1993. Noise exposure was defined on a job-exposure matrix based on a survey of the working conditions carried out during the mid 1970s. All workers were categorised into three main regions of Sweden, differing in temperature: Reference (Götaland), colder (Svealand), and coldest (Norrland). Relative risks (RR) were analysed by negative binomial regression adjusting for age, BMI, and smoking habits.

**Results:**

Moderate and high noise exposure was associated with increased risk of myocardial infarction (RR 1.10–1.13 with 95% CI over unit) and stroke mortality (RR 1.15 to 1.19 with 95% CI over unit). There was an increased risk for myocardial infarction (RR 1.10, 95% CI 1.01–1.20), but not for stroke mortality (RR 1.09, 95% CI 0.94–1.25) associated with living and working in the coldest region. There was an interaction on the risk of myocardial infarction mortality between different regions and noise exposure (*p* = 0.016), but not for stroke mortality (*p* = 0.88).

**Conclusions:**

The study indicates an interaction between working at hazardous noise levels and living and working in cold conditions for increased mortality in myocardial infarction.

**Electronic supplementary material:**

The online version of this article (10.1007/s00420-019-01513-5) contains supplementary material, which is available to authorized users.

## Introduction

Some studies indicate that occupational noise has non-auditory effects on the cardiovascular health such as increased risks of hypertension, coronary heart disease, and stroke (Dzhambov and Dimitrova 2016; Skogstad et al. 2016; Theorell et al. 2016). Several studies have found that living in cold climate or cooling of the body increases mortality in cardiovascular disease (Analitis et al. 2008; Gasparrini et al. 2015; Song et al. 2017). To our knowledge, there is no study on a possible interaction of occupational noise and working in cold conditions on the risk of cardiovascular disease. It is suggested that occupational noise acts as a stressor on the autonomic and endocrine system, which affects risk factors for cardiovascular disease such as increased blood pressure or increased blood viscosity (Basner et al. 2014; Walker et al. 2016). The underlying mechanisms between cold temperatures and mortality are not entirely clear. Cold stress activates the sympathetic nervous system and the endocrine system, and may cause cardiovascular stress by increasing blood pressure, blood viscosity, and vasoconstriction (Analitis et al. 2008; Gasparrini et al. 2015). Cold exposure in laboratory setting is associated with increased blood pressure and heart rate changes, but the effects depend on the type of cooling, part of body cooled, and individual factors (Emmett 1995; Korhonen 2006). Working in extreme cold, such as in cold storage units, is associated with hypertension (Kim et al. 2003).

The aim was to study a possible association between occupational exposure to noise, working and living in cold conditions, and the risk of mortality in myocardial infarction and stroke. Construction workers in Sweden are exposed to hazardous noise levels and they work in cold conditions during winter season, thus are well suited to include in a study on occupational noise, working in cold conditions and the effect thereof on cardiovascular disease.

## Methods

### Study design and population

This cohort study consists of workers in the Swedish construction industry that participated in health examinations between 1971 and 1993. The analysis was restricted to men with normal blood pressure at the first health examination.

The health examinations were offered freely and were performed at an interval of 2–5 years by the nationwide occupational health service up until 1993 (Toren et al. 2007). All construction workers were invited to the health examinations and at least 80% of these workers participated at least once.

There are 389,132 workers in the cohort and 19,418 of them are female. There were few women with high noise exposure (*n* = 99) and 30% of the women had no defined exposure level. Therefore, it is not feasible to include women in the analysis.

Workers below 15 or above 67 years of age at their first health examination were excluded. The retirement age in Sweden was 67 years until 1976 when it was decreased to 65 years.

During the health examinations between 1971–1974 and 1988–1993, the workers answered a questionnaire regarding their working conditions and their health status. Data collected from the health examinations included the region where the examination took place and occupational title. Individual data such as height, weight, tobacco use, and blood pressure were also gathered.

The BMI for all participants were calculated by dividing the weight with the square of the participant’s height. The BMI categories used were 18.5 ≤ BMI < 25 and 25 ≤ BMI < 35 kg/m^2^. Construction workers with BMI over 35 or under 18.5 were excluded, as were workers with missing data for calculation of BMI.

Smoking habits at the first health examination were categorised as “non-smoker”, “previous smoker”, “smoker up to 14 cigarettes per day”, and “smoker with more than 15 cigarettes per day”. If there was no information on smoking habits at the first health examination then this information was retrieved from later examinations if possible.

The blood pressure level was calculated by averaging the blood pressure level at the construction workers’ first health examination and the blood pressure levels at health examinations the following 5 years. If there was no information on blood pressure from the first health examination then the blood pressure measured at a later health examination was used. High blood pressure was defined as 140 mmHg or more in systolic blood pressure, or 90 mmHg or more in diastolic blood pressure.

A job exposure matrix was developed for the 21 different work groups in the cohort. The noise exposure levels were based on a survey of working conditions carried out during the mid-1970s by industrial hygienists. A noise exposure category was assigned for each working group in the cohort. The noise categories were graded on a 1–5 scale. Noise category level 1–3 was at that time considered acceptable noise exposure with noise exposure levels of 45–75 dB(A). At level 4 the noise exposure range was 76–85 dB(A) and for level 5 the noise exposure was above 85 dB(A). For the analysis the noise category levels were categorised as low [≤ 75 dB(A)], moderate [76–85 dB(A)], and high [> 85 dB(A)].

There were 12 different regions in Sweden where the construction workers had their first health examination. These regions were categorised into three main regions of Sweden, representing reference (Götaland), colder (Svealand), and coldest region of Sweden (Norrland). The number of days per year with a daily mean temperature (and standard deviation) below 0 °C from 1970 until 2010, for each region, were on average 67 (21), 80 (18), and 133 (13) days based on data from the Swedish Meteorological and Hydrological Institute (SMHI) (SMHI Open Data [Internet]. Swedish Meteorological and Hydrological Institute (SMHI).[cited 2016 Dec 7]. Available from: https://www.smhi.se/en).

There was a linkage with the National Cause of Death Register to identify which individual had died (underlying cause) from ischemic heart disease (ICD-8410-412, ICD-9410-412, and ICD-10I21-I25) or cerebrovascular disease (ICD-8430-438, ICD-9430-438, and ICD-10I60-I69) using the unique personal identity number.

### Statistical analysis

After excluding workers that did not fulfil the age and BMI inclusion criteria, and after excluding women, there were 359,460 men included in the analysis. Of these men, there were 194,499 with normal blood pressure. Table S1 in online supplementary material presents the exclusion of workers before the analysis.

Negative binomial regression was used to quantify any association between the exposure (noise and cold conditions) and the studied outcomes (myocardial infarction and stroke) among workers with normal blood pressure. Negative binomial regression is an alternative to using quasi-Poisson regression for over-dispersed count data and is similar to Poisson regression. Model performance was assessed using *χ*^2^-tests on the model residuals, and normal QQ-plots to spot any deviance from the normal distribution.

For the analysis of myocardial infarction and stroke, person-years were calculated from inclusion to the cohort to event or censoring. A person was censored when they died, emigrated, or turned 85 years old, or at the end of follow-up in 2010.

Potential confounders included in the analyses were age, BMI, and smoking status gathered at the workers’ first health examination. For the analysis of an association between myocardial infarction and stroke mortality with noise exposure we adjusted for cold exposure using region. When analysing the association of myocardial infarction and stroke mortality with cold exposure we used the three main regions as exposure variable and adjusted for noise exposure. To keep track of changes in the association between exposure and outcome, the initial model was unadjusted and then the potential confounders were introduced individually. The final model included all potential confounders. A possible interaction of noise and region (cold exposure) on mortality of myocardial infarction and stroke was analysed by including an interaction term in the final adjusted model.

Relative risk (RR) and 95% confidence intervals were used to determine statistical significance. For the interaction terms, Analysis of variance (ANOVA) was used to determine statistical significance, with *α* = 0.05. All statistical analyses were performed using R Statistical software (R version 3.4.0).

## Results

At the construction workers’ first health examination and the following 5 years, 63% had normal blood pressure (Table 1). Among the construction workers with normal blood pressure, 39% worked and lived in the colder region of Sweden and 13% in the coldest region (Table 1). Twenty-eight percent of the construction workers had been exposed to high level of noise [> 85 dB(A)] and 44% on moderate levels of noise [76–85 dB(A)]. The workers had similar mean age and BMI between climate regions and noise categories, while there was some differences in smoking habits between regions as presented in online supplementary material, Table S2.

**Table 1:** Descriptive statistics of 194 501 male construction workers with normal blood pressure in the cohort

	*N*	%
Year of birth		
≤ 1910	1269	0.7
1911–1920	8004	4.1
1921–1930	14,818	7.6
1931–1940	25,017	12.9
1941–1950	46,543	23.9
1951–1960	40,364	20.8
1961–1970	51,901	26.7
1971–1980	6585	3.4
BMI category		
Normal (≥ 18.5–25 kg/m^2^)	142,122	73.1
Overweight (≥ 25–35 kg/ m^2^)	52,379	26.9
Smoking habits		
Non-smoker	88,895	45.7
Former smoker	26,789	13.8
Smoker at enrolment (< 15 cig/day)	45,134	23.2
Smoker (≥ 15 cig /day)	33,683	17.3
Region		
Reference	93,812	48.2
Colder	74,931	38.5
Coldest	25,756	13.2
Missing	2	0
Noise		
≤ 75 dB(A)	26,779	13.8
76–85 dB(A)	84,829	43.6
> 85 dB(A)	54,480	28.0
Missing	28,413	14.6

Myocardial infarction and stroke mortality was increased with both moderate [76–85 dB(A)] and high noise exposure [> 85 dB(A)], RR 1.10–1.13 and 1.15–1.19, respectively (Table 2). Myocardial infarction was also significantly increased in the coldest region RR 1.10 (95% CI 1.01–1.20), as seen in Table 3. Noise exposure and climate region of Sweden interacted on the risk of myocardial infarction (*p* = 0.016) but not on stroke mortality (*p* = 0.88), as seen in Table 4. The highest risks were in groups with high noise level and working and living in a cold climate. The highest relative risk of myocardial infarction was in the coldest region for those with the highest noise exposure (Table 4).

**Table 2:** Mortality from myocardial infarction and stroke of male construction workers with normal blood pressure in different noise exposure groups

Models	Exposure		Myocardial infarction			Stroke		
	Noise	*N*	Cases	RR	95% CI	Cases	RR	95% CI
Crude	≤ 75 dB(A) (ref)	26,779	954	1.0		249	1.0	
	76–85 dB(A)	84,829	3210	1.23	0.86–1.75	867	1.54	0.98–2.41
	> 85 dB(A)	54,480	1943	1.21	0.85–1.73	534	1.57	1.00–2.48
Adjusted^a^	≤ 75 dB(A) (ref)	26,779	954	1.0		249	1.0	
	76–85 dB(A)	84,829	3210	1.10	1.01–1.19	867	1.15	1.01–1.32
	> 85 dB(A)	54,480	1943	1.13	1.03–1.23	534	1.19	1.03–1.38

^a^Adjusted for age, BMI, smoking habits, and region

**Table 3:** Mortality from myocardial infarction and stroke of male construction workers with normal blood pressure in different cold conditions

Models	Exposure		Myocardial infarction			Stroke		
	Region	*N*	Cases	RR	95% CI	Cases	RR	95% CI
Crude	Reference	93,812	2856	1.0		767	1.0	
	Colder	74,931	2405	0.96	0.68–1.35	654	0.97	0.64–1.46
	Coldest	25,756	846	0.93	0.65–1.32	229	0.86	0.55–1.36
Adjusted^a^	Reference	93,812	2856	1.0		767	1.0	
	Colder	74,931	2405	0.99	0.93–1.06	654	0.99	0.90–1.10
	Coldest	25,756	846	1.10	1.01–1.20	229	1.09	0.94–1.25

^a^Adjusted for age, BMI, smoking habits, and occupational noise

**Table 4:** Interaction analysis of the different regions of Sweden and noise exposure levels on mortality from myocardial infarction and stroke of male construction workers with normal blood pressure adjusted for age, BMI and smoking habits

	Noise	Region	Myocardial infarction		Stroke	
			RR	95% CI	RR	95% CI
Main effects	≤ 75 dB(A) (ref)		1		1	
	76–85 dB(A)		1.03	0.92–1.17	1.16	0.95–1.42
	> 85 dB(A)		0.97	0.85–1.1	1.16	0.94–1.44
		Reference	1		1	
		Colder	0.89	0.76–1.03	0.98	0.76–1.28
		Coldest	0.86	0.69–1.08	1.10	0.75–1.6
Interaction	≤ 75 dB(A) (ref)	Reference	1		1	
Effects	76–85 dB(A)	Colder	1.07	0.90–1.28	1.00	0.75–1.35
	> 85 dB(A)	Colder	1.28	1.06–1.54	1.02	0.74–1.4
	75–85 dB(A)	Coldest	1.26	0.98–1.63	0.92	0.59–1.42
	> 85 dB(A)	Coldest	1.46	1.12–1.89	1.09	0.7–1.69

## Discussion

The study supports an interaction of occupational noise and living in cold regions, with increased mortality due to myocardial infarction. There was no support for an interaction with stroke. Furthermore, this study supports an association of moderate and high levels of occupational noise and myocardial infarction and stroke. Moreover, a relationship between cold temperatures and myocardial infarction was also found.

Previous studies show an association between occupational noise and cardiovascular diseases and studies showing association with living in cold climate and cardiovascular diseases (Analitis et al. 2008; Dzhambov and Dimitrova 2016; Eriksson et al. 2018; Gasparrini et al. 2015; Skogstad et al. 2016; Song et al. 2017; Theorell et al. 2016). However, to our knowledge this is the first study that shows an interaction between occupational noise, cold temperatures and cardiovascular diseases. Common mechanisms for cardiovascular disease from both occupational noise and cold exposure make an interaction of these exposures increase credibility of the finding.

This study has several advantages. It has a prospective design and a large number of workers, leading to rather high power for the analysis of cold and noise exposures association and interactions. The workers came from the same industry, which limits the influence of socioeconomic and lifestyles factors. There is also information on relevant confounders such as blood pressure, BMI, and smoking habits. The outcomes for this study were retrieved from register data excluding reporting bias.

There are also some limitations to this study. The noise exposure level and the region defined for each worker were collected from their first health examination. The workers could have later on changed their occupation and thereby their noise exposure level or region where they work. The job exposure matrix is constructed from field measurements but for each job title, there were only information on the mean value for the noise exposure. Some individuals will have had lower or higher noise levels than the mean noise level and this could underestimate the risks for myocardial infarction and stroke. The study lacks information on the use of hearing protectors. Hearing protectors were lacking, or, if they were available, were seldom used by the workers in the first half of the twentieth century. There is no information on noise exposure during leisure time, e.g., from traffic noise. There is no information on how much time the workers may have spent outdoors, during work and leisure time, and there is no information on outdoor activities, such as cross-country skiing, snow racing, or shovelling snow, or the use of protective clothing that effect their cold exposure. Most of the misclassifications are probably non-differential which may decrease the associations between occupational noise and/or cold conditions on mortality in cardiovascular diseases. There was no adjustment for alcohol consumption nor leisure time physical activity in the analysis although they are established risk factors for myocardial infarction and stroke. However, it is unlikely that alcohol consumption or leisure time physical activity should vary in relation to noise level or region. A potential bias on effect estimates due to lack of information on these two lifestyle factors is therefore limited.

We only studied men as we had too low power for studying the risk in women, so the risks cannot be automatically transferred to women.

The results were done on a cohort from 1970s until 1993. Today the use of hearing protectors, and the quality of these protectors, has increased over the decades so that the noise exposure into the workers ears has decreased. This may decrease the risk of myocardial infarction and stroke mortality from occupational noise among present day workers. However, the exposure to cold has not changed although there is advancement with clothing; construction workers are still spending much of their work hours outdoors.

## Concluding remarks

The study indicates an interaction between working at hazardous noise levels and living and working in cold conditions for increased mortality in myocardial infarction.

## Electronic supplementary material

Below is the link to the electronic supplementary material.

Supplementary file1 (DOCX 14 kb)
