# Supplementary material for: Occupational exposure to noise and cold environment and the risk of death due to myocardial infarction and stroke
Source: Int Arch Occup Environ Health. 2020 Jan 8;93(5):571–5. doi: 10.1007/s00420-019-01513-5 (PMC7260257; doi:10.1007/s00420-019-01513-5)
Supplement: Supplementary file 1 — Supplementary file1 (DOCX 14 kb) [file 420_2019_1513_MOESM1_ESM.docx]

**Title:** Occupational Exposure to Noise and Cold Environment and the Risk of Death Due to Myocardial Infarction and Stroke

**Journal name:** International Archives of Occupational and Environmental Health

**Authors name and affiliation:** Hans Pettersson, PhD^1^, David Olsson, PhD^1^, Bengt Järvholm, MD^1^

^1^Department of Public Health and Clinical Medicine, Occupational and Environmental Medicine, Umeå University, 901 87 Umeå, Sweden

**Email:** hans.pettersson@umu.se

**Supplement**

**Table S1.** Exclusions of workers in the cohort before analysis.

| Exclusions |  |
| --- | --- |
| All workers in the cohort | 389 132 |
| 19 418 women excluded | 369 714 |
| 350 excluded with age below 15 or above 67 years  at their first health examination | 369 364 |
| 9 904 excluded with BMI under 18.5 or over 35 kg/m^2^  at their first health examination | 359 460 |
| 76 160 excluded since they lack information on smoking habits | 283 300 |
| 164 961 excluded with mean systolic blood pressure of 140 mm Hg or more or mean diastolic blood pressure of 90 mm Hg or more at their first and following five years health examinations. | 194 501 |

**Table S2.** Descriptive statistics of male construction workers with normal blood pressure in the cohort.

| Region of first examination | N | % | Mean age (SD) | Mean BMI  (SD) | Smoker/former smoker N (%) | Myocardial infarction mortality N (%) | Stroke mortality  N (%) |
| --- | --- | --- | --- | --- | --- | --- | --- |
| Reference | 93 812 | 48.2 | 30.6 (11.1) | 23.5 (2.7) | 52 774 (56.3) | 3111 (3.3) | 901 (1.0) |
| Colder | 74 931 | 38.5 | 30.2 (10.9) | 23.7 (2.7) | 39 662 (52.9) | 2093 (2.8) | 613 (0.8) |
| Coldest | 25 756 | 13.2 | 31.9 (11.0) | 23.7 (2.7) | 13 169 (51.1) | 1014 (3.9) | 292 (1.1) |
| Noise |  |  |  |  |  |  |  |
| ≤75dB(A) | 26 779 | 16.1 | 31.6 (11.5) | 23.4 (2.7) | 14 874 (55.5) | 902 (3.4) | 251 (0.9) |
| 76-85 dB(A) | 84 829 | 51.1 | 31.1 (10.8) | 23.7 (2.7) | 46 779 (55.1) | 2806 (3.3) | 818 (1.0) |
| >85 dB(A) | 54 480 | 32.8 | 30.4 (11.1) | 23.6 (2.7) | 29 012 (53.3) | 1771 (3.3) | 535 (1.0) |
